# Introduction: histories of asylums, insanity and psychiatry in Scotland

**DOI:** 10.1177/0957154X16678566

**Published:** 2016-12-12

**Authors:** Chris Philo, Jonathan Andrews

**Affiliations:** University of Glasgow; Newcastle University

**Keywords:** Historiography of psychiatric history, history of psychiatry, Gartnavel Royal Asylum, Royal Edinburgh Asylum, Scotland

## Abstract

This paper introduces a special issue on ‘Histories of asylums, insanity and psychiatry in Scotland’, situating the papers that follow in an outline historiography of work in this field. Using Allan Beveridge’s claims in 1993 about the relative lack of research on the history of psychiatry in Scotland, the paper reviews a range of contributions that have emerged since then, loosely distinguishing between ‘overviews’ – work addressing longer-term trends and broader periods and systems – and more detailed studies of particular ‘individuals and institutions’. There remains much still to do, but the present special issue signals what is currently being achieved, not least by a new generation of scholars in and on Scotland.

## A sparse historiography?

In spite of the wide interest currently shown in the history of psychiatry, little attention has been directed towards events in Scotland. … The lack of detailed research is somewhat surprising, when one considers that the Scottish contribution to the development of psychiatry has been substantial. (Beveridge, 1993: 453)

Introducing his 1993 review of an edited collection on the history of Gartnavel Royal Hospital, Glasgow (Andrews and Smith, 1993), Allan Beveridge lamented the relatively sparse scholarship on the history of asylums, insanity and psychiatry in Scotland. Nearly a quarter-century has now elapsed, and the field has begun to be better cultivated by medical historians, historical geographers and students of architecture, landscape and various other specialist subject-matters.^1^ At least 30 full papers on Scottish themes have since been published in *History of Psychiatry*; a few book length treatments have appeared on periods (e.g. Houston, 2000), psychiatrists (e.g. Barfoot, 1995; Beveridge, 2011), professionals (Andrews, 1998a), pathologies (Davis, 2008) and patients (Berkenkotter, 2008); while a healthy crop of PhD theses have also emerged (Darragh, 2011; Donoho, 2011; Halliday, 2003; McGeachan, 2010; Morrison, 2014; Park, 2007; Ross, 2014; Sturdy, 1996; also two earlier theses: Rice, 1981; Thompson, 1984). It remains the case, however, that Scottish psychiatry’s history is a story still only very partially told;^2^ indeed, it has often been obscured in more broadly conceived historical narratives of British psychiatry. A 1991 issue of *History of Psychiatry* contained several country-based surveys of psychiatric history, including some focused on relatively small European nations (Austria, Belgium, Sweden, Switzerland), but the survey on Britain (Porter, 1991) made no special reference to the Scottish situation. In 2010 the journal carried an assessment of British psychiatry circa 1900 (Freeman, 2010) which tended to conflate ‘Britain’ and ‘England’, excepting brief reflection on why Scotland might have placed a higher value on education than England, leading to the establishment of ‘a psychiatric chair’ at the University of Edinburgh earlier than in any English institution (p. 318). More trenchantly, Andrew Scull has asserted that ‘English-centred historiography of the 1970s and 1980s largely neglected the very different Scottish approaches to the containment and treatment of the mad’ (Scull, 2011: 401).

To underline the importance of what Beveridge called ‘the Scottish contribution’ and also to profile some cutting-edge research that is currently being undertaken on Scottish themes, notably by a new generation of early career scholars, the idea emerged for a special issue of *History of Psychiatry* dedicated to Scottish materials. While the recitation of a given world region’s psychiatric history may hold some intrinsic interest for regional specialists, the guest editors conceive that ultimately its wider import can only be judged in a more analytical and comparative perspective. Arguably, then, when focusing on Scottish psychiatry, at issue should be less the mere historical specificity or importance of the national context *per se* and those rather tired questions about national pre-eminence and distinction – where was a treatment or institution first introduced? or what was different about happenings here rather than there? Rather, we should ask what studies of the Scottish contribution can reveal about broader patterns in a pan-European, or geographically and conceptually wider, transformation of ideas and practices impinging on the mental health dimensions of human vulnerability. Questions about origination and distinctiveness are not irrelevant, of course, but arguably matter most when set in a broader framing of how ideas and practices travel between places, being applied, ignored, inflected or distorted, and likely creating new landscapes of either care and cure or neglect and stigma. This orientation is echoed in all the eight papers that follow: the immediate Scottish locations for each paper are significant, treated with archival and cultural respect, but each one widens into concerns, implications and challenges that rebound beyond Scotland to illuminate much larger terrains of asylums, insanity and psychiatry (past, present and future).

## Overviews (trends, periods, systems)

Until recent years, practically the only guide to the field was D.K. Henderson’s *The Evolution of Psychiatry in Scotland*, a book which perhaps more accurately could have been titled, ‘The Doctor Remembers’, based, as it was, on personal anecdote and reminiscence. (Beveridge, 1993: 453)

In 1964 Sir David Kennedy Henderson, a leading Scottish psychiatrist with an impressive domestic and international pedigree in the mental health sector, published his monograph on the history of psychiatry in Scotland which, as he acknowledged, drew ‘on my memory and experience’ over six decades (Henderson, 1964: vi). While open to charges of partiality of recall and interpretation, his much-read book has since seen no obvious replacement outlining how mental health reform proceeded in Scotland from the eighteenth century through to the 1960s. Henderson’s is no introverted account, narrowly closed around Scotland, but consistently sets Scottish trends within wider worldly currents, stressing substantial international exchanges of ideas and practices, doubtless reflecting his own European and transatlantic connections (Morrison, this issue). The first substantive chapter in the 1964 book, for instance, bears the curious title of ‘Franco-Scottish foursome’, expressly linking together the well-known French lunacy pioneers Pinel and Esquirol with two perhaps less familiar Scotsmen, Andrew Duncan (1744–1828) and Alexander Morison (1799–1866). It might even be objected that there is actually too *little* of Scotland in the book, given the extent to which Henderson digresses into his own assessment of different mental states, institutional settings, research facilities and policy frameworks, incorporating cases, events, texts and authorities from across Britain and beyond.

Henderson (1964: 41) says virtually nothing about the early history of madness in Scotland, other than to state that all ‘previous methods of treatment’, prior to the eighteenth century, ‘were of a crude, rough and ready nature’. He mentions the whipping of the mad, and how ‘many were incarcerated in prisons with criminals and evil-doers’ (p. 42). A more nuanced account of pre-modern madness in the ‘Celtic lands’ of early Britain is given in Basil Clarke’s text *Mental Disorder in Earlier Britain* (Clarke, 1975: esp. ch. 2.1), although even here the specifically Scottish evidence is limited. Intriguingly, though, there are hints at a complex and variegated ‘folklore’ and ‘folk medicine’ around madness, its causes and treatments, arising in ‘the Gaelic areas’ (p. 141) and often connected to natural features such as Scottish lochs, springs and coastlines (e.g. pp. 105–9, 129–33, 105–9). Emily Donoho (2011) digs much deeper into the folk geographies of madness in the Scottish Highlands and Islands, suggesting how in effect a pre-modern world drenched in ‘supernatural madness’ collided with the modern world of lunacy reform occasioned by the coming of the lunatic asylums to northern Scotland from the 1860s. The result, she writes, created ‘a fissured and chaotic psychiatric landscape’, one ‘arguably traumatised by a collapsing of several hundred years of psychiatric history elsewhere’ – a history of ‘“peasant” traditions *gradually* supplanted by asylums, physicians and medico-juridical procedures’ – instead here compressed ‘into a mere half-century or so’ (p. 338, original italics).

Donoho also stresses the role of local legal proceedings in ascertaining the madness or otherwise of troubled Highlanders, showing how families could be quite instrumental in using the sheriffs’ courts to have their mad kinsfolk removed to the care of appointed guardians or even to the asylums. Such an orientation dovetails with R.A. Houston’s ground-breaking monograph *Madness and Society in Eighteenth-Century Scotland* (Houston, 2000; see also Houston, 2001a, 2001b), which trawls copious local sources, especially civil court inquests (‘brieves’) concerning people whose mental capacity was in question, to reconstruct everyday understandings of and responses to madness. Harking back to different conceptual and political strains of writing on the history of psychiatry, and alert to swathes of scholarship on other times and places, Houston does more than anyone to set his Scottish inquiries on that wider stage demanded above. For instance, he concludes from his eighteenth-century research that ‘a generalised fear of the insane [does not] seem to have existed in the folk belief of Scotland (or England), as it did in parts of continental Europe’ (Houston, 2000: 393). Similarly, he challenges any simplistic sense of a transition across the century from traditional hostility to Enlightenment toleration, preferring instead to argue that eighteenth-century Scots were well able to distinguish madness from reason – balancing the two fairly and usually non-prejudicially – ‘in a rather precise way which shows considerable discrimination on the part of the assessors’ (p. 399).

Returning to Henderson, it is clear that the key spaces in his history are the ‘royal asylums’ (charitable institutions each with a royal charter) which arose in several Scottish urban centres from the eighteenth century, followed by the ‘district asylums’ (publicly administered and funded, serving amalgams of parishes) from the 1860s onwards. Parallel outline accounts of the royals and the districts surface in histories of Scottish philanthropy and social welfare (Checkland, 1980: ch.9; Ferguson, 1948: 271–84). Further mention will be made shortly of work on particular institutions, but it can be noted that in general the royals have attracted more attention: they were the more glamorous cousins, better resourced, taking wealthier and more educated patients, often sites of therapeutic advance associated with more prominent physicians, and with superior archives and libraries (Bunch, 1975: ch. 7). The extent to which they comprised a discrete unitary sector is moot, and in this respect the districts - and the district lunacy boards - more obviously comprised such a sector, impelled by the letter of late-1850s legislation, and underpinned by a range of explicitly articulated organizational, therapeutic and environmental logics (Ross, 2014, 2017). Lunatic asylums of various stripes nonetheless did appear across Scotland, if unevenly, solidifying into an overall national system, at least in the sense of being overseen from the late-1850s by a dedicated Scottish Lunacy Commission (Andrews, 1998a), tasked with inspecting, reviewing and, where possible influencing, the conditions of the insane wherever they might be found.

The impression might hence be of a nineteenth-century will among prominent Scottish lay and medical authorities substantially to institutionalize the mentally unwell along the lines of what occurred south of the border, but Houston (2014: 304) appends this caveat:

England used to be seen as the norm for the development of institutional care; in fact, it followed only one of several pathways and Wales, Ireland and Scotland were different. … Scotland’s ‘mixed economy of welfare’ had more domestic care and a prominent voluntary sector; like Wales, it had few private asylums.

Indeed, it is sometimes emphasized that Scotland significantly mitigated the resort to institutionalization by investing in a practice of ‘boarding-out’ lunatics, consigning them to the ‘domestic care’ of appointed guardians, individually or sometimes in twos or threes, particularly in the remoter localities (Sturdy, 1996; Sturdy and Parry-Jones, 1999). It is known that Scottish poor relief, both before and after the New Scottish Poor Law of 1845, relied less on institutional forms than was true in England. At its height in the 1880s and 1890s, the care of single patients in private dwellings represented 15–25% of officially registered insane, compared with no more than 8–16% in English and Welsh counties (Andrews, 1998a: 40; Sturdy, 1996: 1, n.1, 91, 356; Sturdy and Parry-Jones, 1999: 86). The Scottish poorhouse was never the English workhouse, either in number or function, and was more ready to persist with ‘outdoor’ support in community settings (Young, 1994). ‘Particular generosity appears to have been shown to the insane, who were not expected to do anything for their own support’, writes an historian of the Old Scottish Poor Law (Mitchison, 2000: 23). If Mitchison somewhat exaggerates the minimal expectations placed upon the capacities and responsibilities of the insane and their families (see, e.g. Houston, 2006), such a charitable orientation arguably shaped parochial responses to lunacy well into the nineteenth century, although cost, classificatory and diagnostic suitability issues undoubtedly fuelled a hesitation to decant pauper lunatics to the royal asylums or even, from the 1860s, to their district brethren. The articulation of Scottish Poor and Lunacy Laws is the subject of the paper by Lauren Farquharson (this issue), where she specifically grapples with the unique sub-system of six ‘parochial asylums’ that emerged in urban West Scotland as sizeable lunatic asylums under the immediate auspices – illegally or at least as legal anomalies – of Poor Law rather than Lunacy Law authorities. These asylums might be caricatured as institutional versions of an essentially *non*-institutional worldview about how best to deal with non-able (-bodied or -minded) paupers.

In the most explicitly Anglo-Scottish comparison in this issue, Gillian Allmond elaborates on the drift of such remarks, as also implied in the discussion of Houston (2000), by wondering about a particularly Scottish emphasis on ‘liberty’: on fostering regimes where the freedom of the person with mental health problems from obviously institutional constraints would be achieved whenever possible. Emphasizing the prevalence of references to ‘liberty’ and ‘freedom’ by the Scottish Lunacy Commissioners, in contrast to their English counterparts, she focuses on the ‘village asylum’ as a Scottish innovation – three district asylums adopted this scheme – which allowed ‘startling openness’ in an institutional layout free of walls, fences, gates and other enclosing spatial forms (see also Halliday, 2003: chs 4 and 6; Ross, 2014: ch. 9).^3^ Leaping forward chronologically, it is evident that a deep concern for liberty, informed by an abiding concern for justice, energized the agitations of the Scottish Union of Mental Patients (SUMP) in the 1970s, arising from Hartwood Hospital (the old Lanark District Asylum), as explored by Mark Gallagher (this issue). Paradoxically perhaps, Vicky Long (this issue; see also Long, 2014) demonstrates that Scotland was tardier than England to embark upon the *de*institutionalization of its mental hospital estate in the wake of political-legislative injunctions from the late-1950s, but her subtle argument is that an engrained de- or non-institutional ethos – possibly with the deeper historical roots considered above – played a part in actually slowing the closure of Scottish mental hospitals. Various hospitals had become the source of ‘care in the community’ experiments beyond their walls, she explains, which meant that, for a period at least, ‘psychiatric deinstitutionalization’ actually reinvigorated, rather than threatened, Scotland’s institutional asylum heritage.

## Individuals and institutions

A general historical overview of Scottish developments … would be very helpful, but much basic research into a host of areas from individuals to institutions would be required before it was possible. (Beveridge, 1993: 454)

In his 1993 comments, Beveridge stated that more ‘basic research’ was needed on specific Scottish themes (presumably he meant sustained archival inquiry into more key features of Scottish psychiatry), before progress might be made towards updating the ‘overview’ given by Henderson (1964). As should be clear, such research has, in some measure, now been forthcoming, not just providing a better basis for reflections on trends, periods and systems, as briefly reviewed above, but also to provide more analytically satisfactory models of the contexts and roles of specific actors, factors and institutions. Reference has already been made to some of the scholarship on institutions, but a degree of arguably inevitable bias may be discerned in the preponderance of work published on specific lunatic asylums, notably the royal asylums with their ‘supremacy’ in the Scottish mental health scene and ‘scope for thought and research on mental states’ (Checkland, 1980: 176). Glasgow Royal Asylum (GRA), Gartnavel, is served by the edited collection published on its 150th anniversary (Andrews and Smith, 1993; see also Andrews and Smith, 1996), covering administrative, architectural, medico-therapeutic, nursing, religious and patient matters, while a follow-up study addresses the situation of its pauper lunatics as efforts were made from the 1870s – as at other royal asylums – to ‘rid’ the GRA of these largely unwanted nuisances (Andrews, 1999). The Royal Edinburgh Asylum (REA), Morningside, has been rather more extensively analysed via a whole host of surveys of its clinicians, patients and their disorders listed in our bibliography, in particular by Beveridge, but also by Michael Barfoot, Carol Berkenkotter, Gayle Davis and Hilary Marland. A decade before this work, a dedicated PhD thesis examined the REA’s origins and running, scrutinizing the imprint of leading superintendent Thomas Clouston, and minutely profiling the socio-medical profiles of private and pauper patients, while a chapter emerging from this study elucidated the management and medico-moral meaning of alcohol at the same asylum (Thompson, 1984, 1988). Since then, further close analysis of patient admissions (1873–1908) to Clouston’s asylum has been conducted (Beveridge, 1995a, 1995b), while a more recent study has elucidated patient death and disposal, the clinical, spatial and moral functions of REA’s ‘dead house’, and wider anatomo-pathological debates (Andrews, 2012).

Dundee Royal Lunatic Asylum (DRLA) has been persuasively placed in its urban-industrial context as less a humanitarian edifice than a response to ‘the economic and social concerns of the early-nineteenth century and the need to emulate the activities of other Scottish towns’ (Walsh, 1999: 195; see also Rice, 1981, 1985; Walsh, 2004). Aberdeen Royal Asylum (ARA) has meanwhile been analysed as exemplifying Victorian psychiatry’s struggles to find efficacious medical interventions that might ‘cure’ patients, while also revealing, ‘in [its] local institutional context, … the characteristics of a moral reformatory’ (Lobban, 1996: 143). Marland (2004) makes substantial use of patient files from the REA in her work on puerperal insanity, and Morag Allan Campbell (this issue) uses DRLA registers and case notes to pose questions about the profiles of women patients receiving this diagnosis, asking whether it tended to be reserved for those women most able to approximate a Victorian ideal of motherhood and effectively debarred for others such as Dundee’s unruly working-class ‘sisterhood’. Campbell expressly cites as an influence Davis’s pioneering work on General Paralysis of the Insane (GPI), syphilis and contemporary sexual politics (Davis, 2008), which draws upon data contained in asylum and laboratory records from GRA, REA and two district asylums, Woodilee (originally Barony Parochial Asylum; see also Gründler’s 2013 work reviewed by Nolte, 2015) and Rosslynlee (originally the Peebles and Midlothian District Asylum; see also Craig, 2008). The Fife and Kinross District Asylum has additionally furnished patient admission data for an epidemiologically-geared study of the insane poor (Doody et al., 1996). There are fewer studies centring on particular district asylums other than ‘in-house’ accounts provided by personally connected psychiatrists or other mental health workers and often commemorating centenaries (for commentary, see Ross, 2014: 42), although Philo (2007) deployed a scalar logic (of region, settings and buildings) to organize a critical study of Craig Dunain (originally the Inverness District Asylum).^4^ Studies also exist touching on specialist Scottish institutions with psychiatric relevance, such as Craiglockhart War Hospital (Webb, 2006) and Jordanburn Nerve Hospital (Davidson, 2009), while Joy Cameron (1983: ch. 7) deals with ‘lunatics in prison’ and the Criminal Lunatic Asylum (then State Mental Hospital) at Carstairs.

The scholarship on people in Scottish psychiatric history has also blossomed. Psychiatrists past and present were name-dropped throughout Henderson (1964), teetering on the edge of a ‘great man’ approach, while Beveridge (1993: 454) advanced ‘William Cullen, Robert Whytt and George Cheyne in the eighteenth century; Alexander Morison, Andrew Combe and W.A.F. Browne in the nineteenth century; and D.K Henderson and R.D. Laing in the twentieth century’ as figures worth special attention. Scull (2011) underlines how various Scots shaped *English* psychiatry, 1700–1980, moving lightly over contributions from, among others, George Cheyne (1671–1743), William Cullen (1710–1790), W.A.F. Browne (1806–1885), Alexander Morison (1799–1866), David Skae (1814–1872), Thomas Clouston (1840–1915), D.K. Henderson (1884–1965) and R.D. Laing (1927–1989). Some were ‘Scots decamping to England’ (Scull, 2011: 408), but there was also traffic in the other direction, as Scull remarks, notably Thomas Laycock (1812-1876), from Yorkshire in England, who lectured on medical psychology in Edinburgh. Another example of Scottish influence travelling south, cast by Roy Porter (1988) as ‘Brunonian psychiatry’, was the ‘excitability’ thesis of Scotsman John Brown (1735–88). Several of these individuals have been researched in more detail elsewhere: Cheyne (Guerrini, 2000; Shuttleton, 1993), Browne (Scull, 1991: Introduction), Skae (Barfoot, 2009; Fish, 1965), Clouston (Beveridge, 1991; O’Connor, 2015) and Laycock (Barfoot, 1995; James, 1998; Philo, 2006); and to this list can be added scholarship on David Yellowlees (1836-1921) (Andrews, 1997a, 1997b). Some of these studies have striven to locate analysis of such individuals in the broader comparative context of regional or metropolitan-hospital and university based ‘schools of psychiatry’, and their interfaces with supra-national psychiatric movements and career paths. Henderson is the fulcrum of Hazel Morrison’s PhD thesis (Morrison, 2014), which explores his deployment of a ‘case conference approach’ at GRA inspired by Meyerian psychobiology. In her paper for this issue she dissects the transatlantic linkages between Henderson, Charles Macfie Campbell (1876–1943, another Scot) and Adolf Meyer, a Swiss émigré in America. Sarah Phelan (this issue) complements Morrison’s work by considering a lesser-known figure, Thomas Ferguson Rodger (1907–78), who worked both under Henderson at GRA and at Meyer’s Phipps Clinic in Baltimore. Phelan’s specific contribution here is to unpack a self-conscious ‘eclecticism’ in Rodger’s theory and practice, reflecting his psychiatric heritage alongside the challenge of post-WWII deinstitutionalization. Rodger might perhaps be construed as a ‘missing link’ between Henderson and Laing, not least because of a lingering adherence to psychoanalysis. Laing himself has been examined in terms of his Glasgow upbringing, his clinical experience at GRA and his wider metropolitan connections, as well as his ‘rumpus room’ experiments and psycho-dynamic therapy (Abrahamson, 2007; Andrews, 1998c; Beveridge, 2011; Collins, 2008; Hunter-Brown, 2008; McGeachan, 2010, 2013a, 2013b, 2014a, 2014b, 2017; Miller, 2004, 2012). Miller (2009), meanwhile, has contemplated the question ‘How Scottish was R.D. Laing?’; while elsewhere (Miller, 2015) he considers the Scotland-psychotherapy couplet through the person of Winifred Rushforth (1885–1983).

The people in the eye of the psychiatric storm are the patients, of course, and various attempts have been made to access the circumstances and experiences of individuals admitted to Scottish mental institutions. Andrews (1998b; see also Walsh, 1999) has paved the way, using GRA case notes and patient histories, and in this special issue he takes the lead in commentating on the issue’s Classic Text, a tract of 1860, *Philosophy of Insanity*, authored by James Frame, a patient at GRA in 1843, and again in 1856. Morrison (2013, 2014) deploys literary and cultural-history lenses to probe patient stories from GRA case conferences convened by Henderson and colleagues, while Berkenkotter (2008) examines REA case histories as part of a broader inquiry into changing forms of case narrative construction, emphasizing not so much the patient ‘voice’ as the shifting psychiatric languages for translating it. The REA archives have also yielded patient letters and other records illuminating the ‘inmate’ world (Barfoot and Beveridge, 1990, 1993; Beveridge, 1998), nudging towards a fuller appreciation of what it was actually like to dwell, socialize, imagine and hope or fear in the everyday spaces of a premier Scottish asylum. Beveridge has also authored a series of pieces on the ‘little madnesses’, or on occasion more full-blown mental disorders, endured by various Scottish poets and writers, covering individuals as diverse as Robert Fergusson, Iain Crichton Smith and Muriel Spark (Beveridge, 1990, 1996, 2016). Taking seriously the writings and artworks of less famous institutionalized patients permits a glimpse into their perceptions, often resentful about wrongful confinement and offering a critical perspective on what one patient at GRA and then CRI, John Gilmour, denounced as ‘The Lunatic Manufacturing Company’ of asylums and alienists (Beveridge and Williams, 2002; see also Baker 1978; Beveridge and Watson, 2006). Superintendent Browne’s collection of patient art at the Crichton Royal Institution (CRI) has proved a rich source for study, notably by Maureen Park (2007, 2010), while Philo (2006) evaluates how the doodles and letters of one CRI patient, the Lakeland artist William Blacklock, were integrated into the psychiatry of the Edinburgh physician Laycock. Cheryl McGeachan (this issue) offers a sample of ongoing research on the ‘Art Extraordinary’ artworks collected from Scottish asylums by Joyce Laing, Scotland’s first psychiatric art therapist. McGeachan here reconstructs what the stone head-carvings by Adam Christie, a patient at the Royal Montrose Asylum (MRA) 1901–50, disclose about Christie’s inhabitation of the ‘small spaces’ of the institution. Mark Gallagher (this issue) considers not the artworks but rather the political works of Thomas Ritchie, patient at Hartwood Hospital and founder of SUMP (see above), which, although in a very different register from Christie’s stonework, also opens a rare window on Scottish psychiatric history as lived ‘from below’.

## Notes

1. For work specifically on the history of learning disability in Scotland, see: Anderson et al., 1997; Egan, 2001, 2006; Hutchinson, 2011; Macmillan, 1998.

2. Moreover, as one of our referees adds: ‘It is not just history of psychiatry that remains underexplored in a Scottish context, it is the history of health and welfare more generally … [I]f one looks even at recent writing on modern Scotland, it is somewhat obsessed with issues of national identity … at the expense of something which actually affected many Scots much more directly, health and welfare’.

3. For more on the architectural and spatial history of Scottish asylums, see: Darragh, 2011; Markus, 1982; Philo, 1999; Richardson and MacInnes, 2010: ch.3.

4. Donoho (2011) makes extensive use of admissions registers for this asylum (see also Souter, 2013) and those of Lochgilphead (originally the Argyll and Bute District Asylum).
